# Overcoming barriers for investigating nickel-pincer nucleotide cofactor-related enzymes

**DOI:** 10.1128/mbio.03404-24

**Published:** 2024-12-16

**Authors:** Jorge L. Nevarez, Aiko Turmo, Santhosh Gatreddi, Swati Gupta, Jian Hu, Robert P. Hausinger

**Affiliations:** 1 Department of Chemistry, Michigan State University, East Lansing, Michigan, USA; 2 Department of Microbiology, Genetics, and Immunology, Michigan State University, East Lansing, Michigan, USA; 3 Department of Biochemistry and Molecular Biology, Michigan State University, East Lansing, Michigan, USA; Freie Universitat Berlin, Berlin, Germany

**Keywords:** nickel, cofactor, racemase, epimerase, circular dichroism spectroscopy

## Abstract

The nickel-pincer nucleotide (NPN) cofactor is a modified pyridinium mononucleotide that tri-coordinates nickel and is crucial for the activity of certain racemases and epimerases. LarB, LarC, and LarE are responsible for NPN synthesis, with the cofactor subsequently installed into LarA homologs. Hurdles for investigating the functional properties of such proteins arise from the difficulty of obtaining the active, NPN cofactor-loaded enzymes and in assaying their diverse reactivities. Here, we show that when the *Lactiplantibacillus plantarum lar* genes are cloned into the Duet expression system and cultured in *Escherichia coli*, they confer lactate racemase activity to the cells. By replacing *L. plantarum larA* with related genes from other microorganisms, this system allows for the generation of active LarA homologs. Furthermore, the Duet system enables the functional testing of LarB, LarC, and LarE homologs from other microorganisms. In addition to applying the Duet expression system for synthesis of active, NPN cofactor-containing enzymes in *E. coli*, we demonstrate that circular dichroism spectroscopy provides a broadly applicable means of assaying these enzymes. By selecting a wavelength of high molar ellipticity and low absorbance for a given 2-hydroxy acid substrate enantiomer, the conversion of one enantiomer/epimer into the other can be monitored for LarA homologs without the need for any coupling enzymes or reagents. The methods discussed here further our abilities to investigate the unique activities of Lar proteins.

**IMPORTANCE:**

Enzymes containing the nickel-pincer nucleotide (NPN) cofactor are prevalent in a wide range of microorganisms and catalyze various critical biochemical reactions, yet they remain underexplored due, in part, to limitations in current research methodologies. The two significant advancements described here, the heterologous production of active NPN-cofactor containing enzymes in *Escherichia coli* and the use of a circular dichroism-based assay to monitor enzyme activities, expand our capacity to analyze these enzymes. Such additional detailed characterization will deepen our understanding of the diverse chemistry catalyzed by the NPN cofactor and potentially uncover novel roles for this organometallic species in microbial metabolism.

## INTRODUCTION

**Fig 1:**
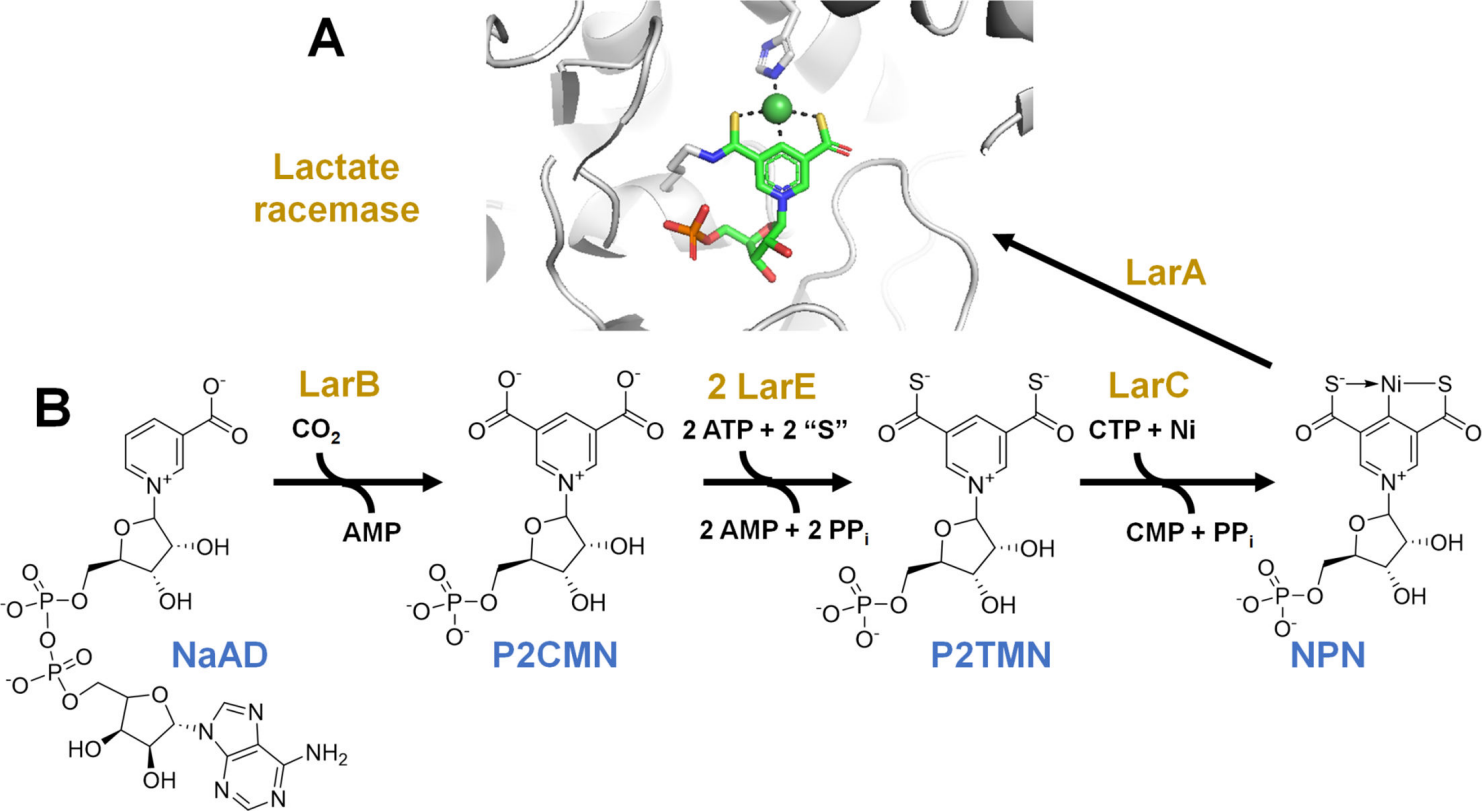
The structure and biosynthesis pathway for the NPN cofactor of lactate racemase. (**A**) Structure of the NPN cofactor in lactate racemase of *L. plantarum* (PDB: 5HUQ). (**B**) Pathway for biosynthesis of the NPN cofactor where “S” is either a cysteinyl sulfur of LarE creating a dehydroalanine residue or a non-core sulfide bound to a tri-Cys coordinated [4Fe-4S] cluster in LarE, depending on the source of the enzyme.

The nickel-pincer nucleotide (NPN) cofactor was first identified in lactate racemase from *Lactiplantibacillus plantarum* (LarA*_Lp_*) and shown to be a pyridinium-3,5-dithiocarboxylic acid mononucleotide with nickel coordinated to the two thiocarboxylate sulfurs and C4 of the pyridinium ring (1). The enzyme provides a histidyl residue to complete the square-planar coordination geometry of the metal ion and a lysyl residue to covalently tether the cofactor by a thioamide linkage (Fig. 1A). The cofactor does not always form an adduct with the enzyme as shown by LarA of *Thermoanaerobacterium thermosaccharolyticum*, which loses the non-covalently bound NPN during enzyme purification (2).

The pathway for biosynthesis of the NPN cofactor is established (Fig. 1B). LarB adds CO_2_ to C5 of nicotinic acid adenine dinucleotide (NaAD) and hydrolyzes the phosphoanhydride to release AMP and produce pyridinium-3,5-dicarboxylic acid mononucleotide (P2CMN) (3, 4). LarE from *L. plantarum* activates one of the P2CMN carboxylic acids by adenylylation while releasing pyrophosphate (PP_i_), forms a covalent adduct with substrate using Cys176 while releasing AMP, and transfers the Cys176 sulfur to generate pyridinium-3-carboxy-5-thiocarboxylic acid mononucleotide (PCTMN) while forming a dehydroalanyl residue (Dha) in the protein (3, 5). Another round of these reactions converts PCTMN to pyridinium-3,5-dithiocarboxylic acid mononucleotide (P2TMN) while hydrolyzing ATP to AMP and PP_i_ and forming Dha176 in a second LarE molecule (6). Alternatively, LarE from organisms such as *Thermotoga maritima* catalyzes similar transformations, but it possesses a [4Fe-4S] cluster bound by three cysteinyl residues that accepts a non-core sulfide at its open iron site from cysteine desulfurase acting on the free amino acid L-cysteine, and this sulfide from the resulting [4Fe-5S] cluster is added to the adenylylated carboxyl groups of the substrates (7). In the final biosynthetic step, *L. plantarum* LarC installs nickel ion into P2TMN to form the NPN cofactor in a CTP-dependent process (8). Studies of LarC from *Moorella thermoacetica* showed that CTP is used to cytidinylylate the phosphate group of P2TMN, with the enzyme hydrolyzing the phosphoanhydride after nickel is incorporated (9).

Bioinformatic analysis of over 1,000 eubacterial and archaeal genomes indicated that homologs of LarA and the NPN biosynthesis pathway enzymes are present in approximately 9% of this population (2). Recent biochemical studies demonstrated that seven out of 13 potential *larA* homologs carried out a reaction distinct from lactate racemization; namely, racemization or epimerization of other 2-hydroxyacid substrates such as malate, 2-hydroxyglutarate, and the sugar D-gluconate (10). Moreover, ~15% of the genomes analyzed contain homologs of *larB*, *larE*, and *larC*, but lack a *larA* homolog, suggesting that some microorganisms, including many cyanobacteria, synthesize the NPN cofactor using the usual pathway, but then incorporate the molecule into one or more non-LarA-like NPN-binding proteins.

Given the widespread appearance and diverse functionality of the LarA homologs and the likely presence of non-LarA-like NPN cofactor-binding proteins, it is important to both (i) develop methods that allow for the generation of cofactor-containing (active holoprotein) forms of these enzymes from diverse microorganisms and (ii) devise routine assays for characterizing the properties of the different 2-hydroxyacid racemases/epimerases. Here, we describe methods to fulfill these needs. Notably, these approaches also can be used to confirm the reactivities and characterize the attributes of LarB-, LarE-, and LarC-like proteins. By adopting these methods to the study of Lar proteins, investigators will be able to better analyze the NPN utilizing and synthesizing enzymes and obtain a more complete understanding of the full catalytic potential of the NPN cofactor.

## MATERIALS AND METHODS

### Gene, plasmids, and cloning

Bacterial strains, plasmids, and primers used for this study are listed in Table S1. PCR amplifications were performed using Q5 high-fidelity DNA polymerase following the manufacturer’s protocol (NEB, Ipswich, MA, USA) or using Vazyme Phanta Flash Super-Fidelity DNA polymerase (Nanjing, China). The primers used were purchased from IDT (Integrated DNA Technologies, Coralville, IA, USA). DNA fragments were ligated using the *in vivo* subcloning assembly method (11, 12) or using Vazyme ClonExpress II One Step Cloning Kit. The transformations were performed using a standard chemical method in *Escherichia coli* DH5α and BL21 (DE3) cells for plasmid amplification and protein expression purposes, respectively (13).

The four *lar*-related genes from *L. plantarum* were incorporated into pETDuet (*larA* and *larB*) or pRSFDuet (*larE* and *larC*) plasmids (Merck KGaA, Darmstadt, Germany) (14) to form pAT035 or pAT038, respectively. The homologs of *larA* from *Megasphaera elsdenii* (10) were exchanged for the version from *L. plantarum*. Similarly, genes encoding LarC of *M. thermoacetica* and homologs of the NPN cofactor biosynthesis enzymes from *Synechocystis* sp. PCC 6803 were switched with *larB*, *larC*, or *larE* of *L. plantarum*. All homologous genes were codon optimized for expression in *E. coli*, chemically synthesized (IDT), and sequentially swapped for the *L. plantarum* genes.

### Purification of *Isosphaera pallida* LarA from *Lactococcus lactis*

The Strep-tagged LarA homolog from *I. pallida* (LarA*_Ip_*, also named LarAH31) was expressed from pGIR210-LarAH31 (constructed from the pBAD-derived vector encoding LarAH2/Sar [10]) in *L. lactis* NZ3900 that was grown with shaking at 30°C in M17 medium supplemented with 0.5% glucose and 7.5 µg/mL chloramphenicol. After reaching an OD_600_ of 0.3–0.4, 1 mM NiCl_2_ and 5 µg/L of nisin A were added, and the culture was grown for an additional 3–4 h. After overnight storage at 4°C, the cells were collected by centrifugation (4,000 × *g* for 15 min), resuspended in 100 mM Tris buffer, pH 7.5, containing 150 mM NaCl, 10 µg/mL lysozyme, and 2 µg/mL of DNaseI, and stirred at 4°C for 1 h. To the suspension was added 1 mM phenylmethylsulfonyl fluoride (PMSF), and cell lysis was carried out by two passes through the French press at 16,000 psi. The supernatant was collected by centrifugation (18,000 × *g* for 70 min). LarA*_Ip_* was purified using StrepTactin XT resin (IBA, Göttingen, Germany) that was equilibrated with 100 mM Tris buffer, pH 7.5, containing 150 mM NaCl. After loading the sample, the column was washed with the same buffer, and then enzyme was eluted with this buffer containing 50 mM biotin and 50 mM NaOH. To improve the sample purity, the concentrated protein was chromatographed on a Superdex 200 increase 10/300 GL column equilibrated with 50 mM Tris buffer, pH 7.5, containing 300 mM NaCl. The monomeric peak fractions from the gel filtration column were used for further studies. Protein concentrations were determined by using a Nanodrop spectrophotometer and calculated with an extinction coefficient of 35,410 M^−1^ cm^−1^ matching the protein sequence.

### Purification of active Strep-tagged LarA homologs from *E. coli*

In addition to using the above established method for purifying LarA homologs from recombinant *L. lactis* cells with nisin induction (15), we developed a new approach to generate active LarA homologs in *E. coli* BL21 (DE3) using the Duet plasmid system (14). The *E. coli* cells that had been co-transformed with pAT035 and pAT038, or their derivatives, were grown in autoinduction medium (16) or terrific broth (TB) medium containing 50 µg/mL kanamycin and 100 µg/mL carbenicillin at room temperature while shaking at 220 RPM to an OD_600_ of 0.6–0.8. The cultures in TB medium were induced with 1 mM isopropyl-D-1-thiogalactopyranoside (IPTG). All cultures were incubated at room temperature for 20–24 h. When indicated, 1 mM of NiCl_2_ and/or 1 mM of nicotinic acid (final concentrations) was added after 4 h of growth. Cell pellets were resuspended in 100 mM Tris, pH 7.5, buffer containing 150 mM NaCl and stored at −80°C until use. Thawed cells were adjusted to contain 0.5 mM Na_2_SO_3_ (for cofactor stabilization), 1 mM PMSF, one tablet of cOmplete EDTA-free protease inhibitor cocktail (Roche, Basel, Switzerland), 1 mM lysozyme, 1 mM dithiothreitol (DTT), and 1 unit of benzonase. The cells were lysed by two passes through a French pressure cell at 16,000 psi. Strep-tagged LarA or its homologs were purified using a StrepTactin XT resin (IBA, Göttingen, Germany) with buffer that included 100 mM Tris at pH 7.5, 300 mM NaCl, and 0.05 mM Na_2_SO_3_, and the proteins were eluted with 50 mM biotin (2). Protein concentrations were determined by the Bradford protein assay reagent (Bio-Rad, Hercules, CA, USA) using bovine serum albumin as the standard.

### Coupled enzyme activity assays

The purified Strep-tagged LarA*_Lp_* protein was buffer exchanged to remove the Na_2_SO_3_ and biotin from the buffer using a PD-10 desalting column (Cytiva, Marlborough, MA, USA). To assess the Lar activity by the conventional assay, LarA*_Lp_* or its homologs (1 pmol) were mixed with 5–400 mM sodium L-lactate in 100 mM 4-(2-hydroxyethyl)-1-piperazineethanesulfonic acid (HEPES) buffer, pH 7.0, for up to 12 min at 35°C, then boiled for 10 min at 95°C to inactivate the enzyme. The precipitated protein was removed by centrifugation at 17,000 × *g*, and the supernatant was collected. The amount of D-lactate in the sample, produced by the lactate racemase activity of LarA, was measured using a commercial kit (Neogen, Lansing, MI, USA) as previously described (2). The same kit was utilized when studying phenyllactate racemase, examining both L-lactate and L-phenyllactate as substrates.

### Circular dichroism spectroscopy-based assay

An alternative assay that can be used with a wide assortment of NPN-containing racemases and epimerases made use of changes in molar ellipticity as monitored by circular dichroism (CD) spectroscopy. This assay was inspired by similar studies involving mandelate racemase (17), which also utilizes a 2-hydroxyacid substrate. The L- or D-2-hydroxyacid sample was scanned while recording both the molar ellipticity and absorbance versus wavelength. By dividing the former by the latter, the wavelength providing the largest signal for molar ellipticity with low noise was identified. Changes in ellipticity at the selected wavelength allowed for real-time monitoring of racemization. The CD spectra were recorded using a Jasco J-815 CD spectrometer equipped with a Jasco CDF-426s/15 temperature control unit. For substrate CD spectra scans, the typical settings were as follows: wavelength range of 200–300 nm, data pitch of 0.5 nm, digital integration time (DIT) of 2 s, scanning speed of 100 nm/min, bandwidth of 1 nm, and three accumulations. For continuous wavelength monitoring, the typical settings included a data pitch of 1.0 s, a DIT of 4 s, and a bandwidth of 1 nm. All measurements were conducted using a 10-mm pathlength quartz cuvette.

The initial rates of LarA*_Lp_* activity were determined by monitoring the change in molar ellipticity at 232 nm. A standard curve was generated using known concentrations of D- and L-lactate, with the slope of the curve defining the relationship between molar ellipticity at 232 nm and lactate concentration. The rate of conversion of one isomer of lactate to the other was described by equation 1.


(1) $$v=\left|\frac{\frac{\Delta \theta }{\Delta t}}{2l\left[\theta \right]}\right|$$

where *v* is the velocity of the reaction, Δ*θ*/Δ*t* represents the rate of change in observed ellipticity, *l* is the sample pathlength, [θ] is the ellipticity of lactate at the monitored wavelength, and the value 2 accounts for the equal and opposite contribution to the total ellipticity by the product.

Initial velocities were determined by substituting the measured values of Δ*θ*/Δ*t* and [θ] into equation 1, with Δ*θ*/Δ*t* being the slope of the linear portion of the reaction plot (from 0 to 12 s of the reaction) for each substrate concentration, and [θ] being 417.15 deg. mol^−1^ cm^2^, the molar ellipticity at 232 nm for lactate. These initial velocities were then plotted against substrate concentrations to generate Michaelis-Menten plots. The data were fitted to the Michaelis-Menten equation using the “curve fit” function from SciPy in Python to obtain *K*_*m*_ and *V*_max_ values. To calculate the catalytic rate constant (*k*_cat_), *V*_max_ was divided by the enzyme concentration (2.1 µM) and converted to s^−1^. Analogous approaches were utilized for monitoring catalysis involving other 2-hydroxyacids.

### Lissamine rhodamine B sulfonyl azide (LRSA) labeling for detection of the protein-bound NPN

To assess whether the LarA homologs had covalently incorporated the NPN cofactor, we investigated the ability of LRSA, a reagent that reacts with thiocarboxylic acids, to react with the protein-bound NPN after dissociation of the nickel, i.e., protein-bound P2TMN (18). Synthesis of LRSA was carried out as previously described (19). The protocol for LRSA labeling of P2TMN-bound proteins was based on and modified from the procedure for labeling proteins that terminate in a thiocarboxylic acid at their carboxyl end (19, 20). For this analysis, 0.5 g of cells was resuspended in 0.7 mL of 100 mM Tris-buffered saline containing 150 mM NaCl at pH 7.5 and was transferred to a 2-mL tube to be lysed with a mini-beadbeater. The lysates were centrifuged, and the supernatant solutions were collected. The samples were roughly normalized based on the overall protein content using the absorbance at 280 nm, and buffer exchanged into 50 mM potassium phosphate, 300 mM NaCl, and 6 M urea, at pH 6.1. To each of the samples was added 10 µL of 15 mM LRSA in dimethyl sulfoxide, and the vials were left to react in the dark at room temperature for 20 min. The protein portions of the samples were precipitated using the chloroform-methanol method (21) and resuspended in the phosphate urea buffer stated above. Each sample (20 µL) was mixed with 5 µL of fivefold concentrated sodium dodecyl sulfate (SDS)-loading buffer and 20 µL was loaded onto a 12% acrylamide gel, subjected to SDS-polyacrylamide gel electrophoresis (PAGE), and used for imaging the proteins with bound LRSA followed by staining with Coomassie brilliant blue. Analogous steps were used when examining purified proteins. The rhodamine-bound gel bands were excited at 530 nm while monitoring the emission at 580 nm and were documented using the ChemiDoc MP imaging system (BioRad, Hercules, CA, USA).

### Ultraviolet (UV)-visible spectroscopy

The spectra of the purified LarA proteins were measured using a quartz cuvette with a Shimadzu UV-2600 spectrophotometer (Kyoto, Japan) at room temperature. Sample volumes were 1 mL.

### Nickel content analysis

Quantification of the LarA nickel content was carried out by using an Agilent 710 Series (Santa Clara, CA, USA) inductively coupled plasma optical emission spectrometer (ICP-OES). The samples were prepared by adjusting to 35% (wt/vol) HNO_3_ and heating at 95°C for 1 h to mineralize the components. A final concentration of 0.1 ppm Yttrium (Sigma-Aldrich, St. Louis, MO, USA) was added to all samples as an internal standard. A nickel standard curve and a buffer control were used to account for background nickel contamination. Data were collected and analyzed using the ICP Expert II software.

### Mass spectrometric analysis

Intact protein masses were analyzed by using a Waters G2-XS Q-TOF (time of flight) mass spectrometer by injecting 10 µL of sample onto a Thermo Hypersil Gold CN guard column (1.0 × 10 mm) for desalting. A gradient was run as follows using 0.1% formic acid in water (solvent A) and acetonitrile (solvent B) at a flow rate of 0.1 mL/min: initial conditions were 98% A/2% B, hold at 2% B to 5 min with the flow diverted to waste for the first 3 min, ramp to 75% B at 10 min and hold at 75% B to 12 min, return to 2% B at 12.01 min and hold to 15 min. Mass spectra were obtained using electrospray ionization in positive ion mode with a source temperature of 100°C, cone voltage of 35 V, desolvation temperature of 350°C, desolvation gas flow of 600 L/h, cone gas flow of 50 L/h, and capillary voltage of 3.0 kV. Data were acquired using a 0.5 s time-of-flight mass spectrometry (TOF MS) scan across an *m/z* range of 200–2,000. The spectra were deconvoluted in Masslynx using the maximum entropy (MaxEnt) I algorithm.

## RESULTS

### Synthesis of active LarA*_Lp_* in *E. coli*

In prior studies, the four *L. plantarum lar* genes were placed under the control of a nisin-inducible promoter and transformed into *L. lactis* lacking the *lar* genes (2). Homologs of *larA* from other microorganisms could then be substituted for the corresponding *L. plantarum* gene within this Gram-positive host resulting in a few active enzymes, but this method was not generally successful due to plasmid instability and other confounding issues (10). In parallel with the *L. lactis* expression studies, earlier efforts expressed individual *larA* homologs in *E. coli*, and the resulting apoproteins were mixed with biosynthetically produced NPN cofactor, a time-consuming and error-prone process that required the isolation and utilization of LarB, LarE, and LarC (Fig. S1) (10).

**Fig 2:**
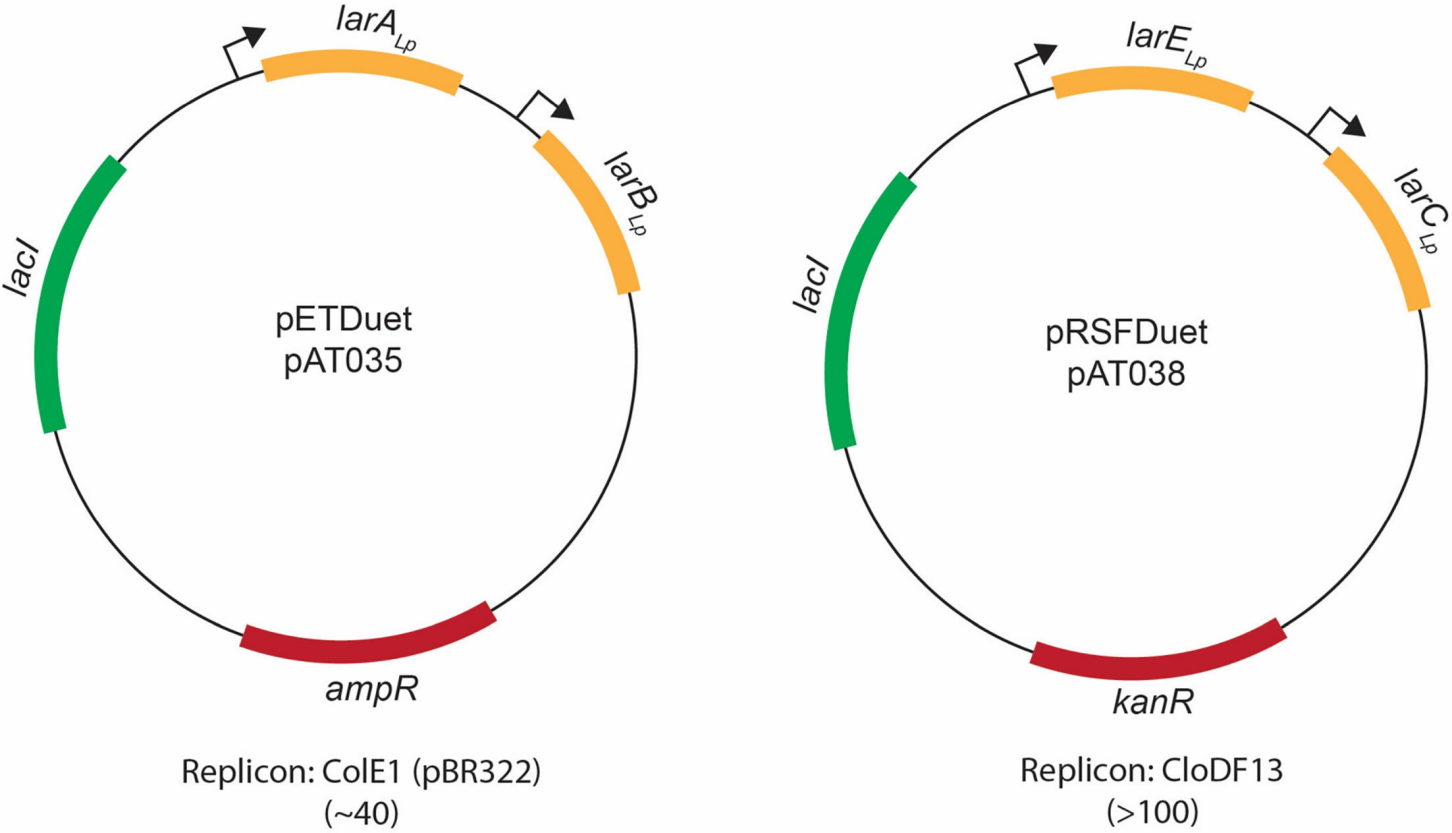
Plasmid design for expression of *lar* genes in *E. coli*. The number in parentheses indicates the plasmid copy number.

Here, we co-expressed the four *lar* genes of *L. plantarum* within *E. coli* using the pETDuet and pRSFDuet plasmids of the Duet vector system (14) and successfully generated lactate racemase activity in this genetically tractable and easily manipulated host microorganism. Importantly, these plasmids are stably maintained in the cells due to their compatible replication origins and different antibiotic resistance cassettes. The genes encoding LarE*_Lp_* and LarC*_Lp_* were selected for expression using the pRSFDuet plasmid (creating pAT038), a high copy number vector, because these proteins are thought to catalyze single turnover reactions, whereas *larA* and *larB* from *L. plantarum* were expressed from the pETDuet plasmid (forming pAT035) (Fig. 2). Importantly, the gene encoding LarA*_Lp_* was cloned with a sequence for a Strep-tag on its C-terminus for easy purification prior to analyzing activity and testing for the presence of covalently bound NPN cofactor. Using this new system, we showed that *larA* homologs from other microorganisms can be swapped for the gene encoding LarA*_Lp_* to obtain NPN-containing forms of those alternative proteins. In addition, we demonstrated that genes encoding homologs to the NPN biosynthetic enzymes can be exchanged for the corresponding genes in this system to test their biosynthetic functionality. This new approach avoids the problems of the *L. lactis* cloning and expression system and the need to purify from *E. coli* each of the individual enzymes required for NPN biosynthesis. As described below, we demonstrated the production of active LarA*_Lp_* by (i) revealing the presence of covalently bound P2TMN using a fluorescent staining procedure that reacts with the novel thiocarboxylic acid, (ii) utilizing UV-visible spectroscopy to identify the chromophore associated with the cofactor, (iii) establishing the presence of nickel in the protein, (iv) validating the expected increase in protein size by mass spectrometry, and (v) directly assessing the enzyme activity.

**Fig 3:**
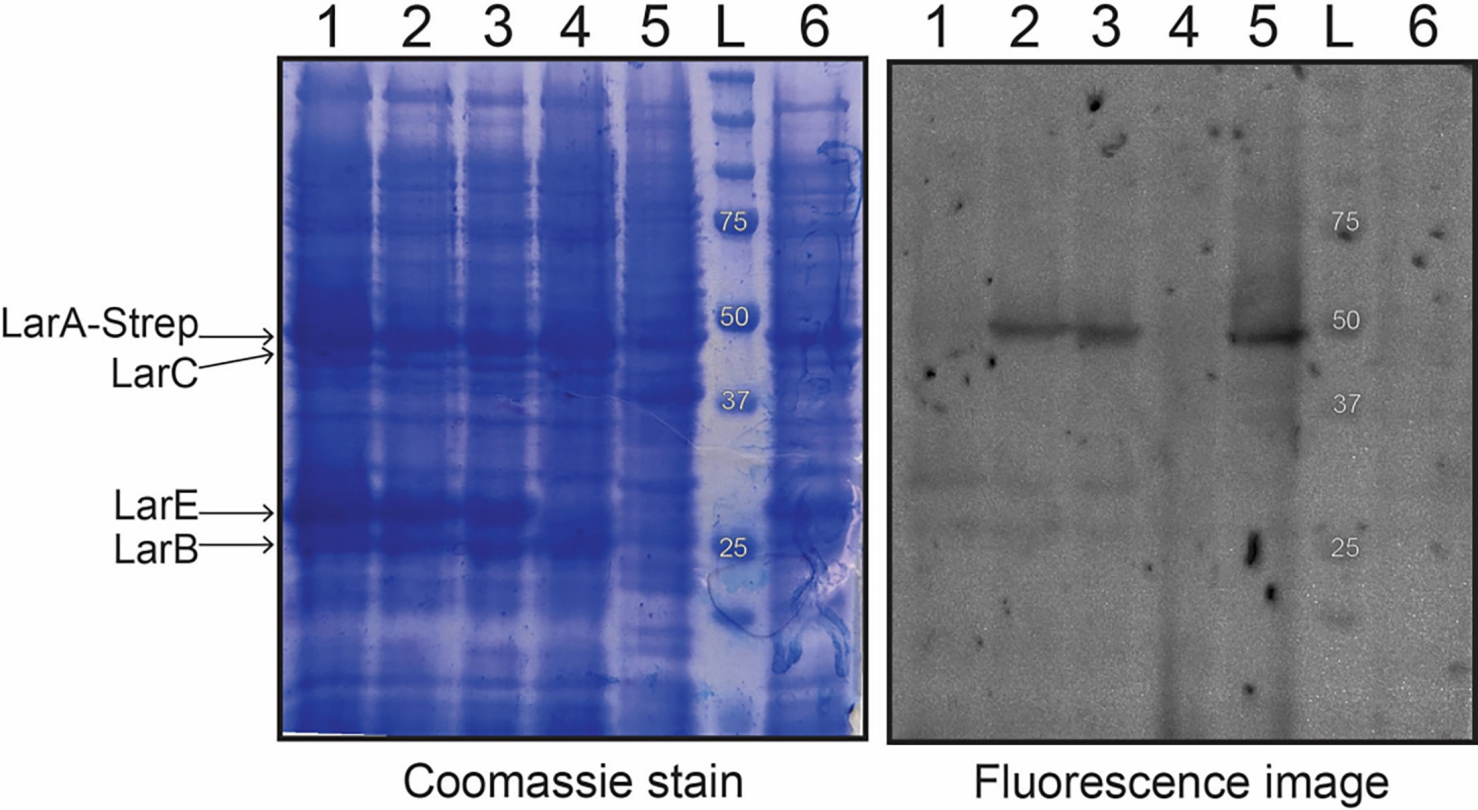
LRSA labeling of NPN-bound protein in cell-free *E. coli* lysates. LRSA-labeled protein samples were subjected to denaturing gel electrophoresis, imaged for fluorescence (right, with excitation and emission wavelengths of 530 and 580 nm), and stained with Coomassie brilliant blue (left). Lane 1: lysate of culture expressing pAT035 and pAT038 that was supplemented with 1 mM nicotinic acid; lane 2: lysate of this culture with 1 mM NiCl_2_; lane 3: lysate of this culture with 1 mM nicotinic acid and NiCl_2_; lane 4: lysate of culture expressing pAT035 alone with 1 mM nicotinic acid and NiCl_2_; lane 5: *L. lactis* (pGIR112) lysate of cells supplemented with 1 mM NiCl_2_ during growth; lane L: protein ladder; lane 6: culture with no additive.

Cell-free lysates derived from the *E. coli* Duet system expressing the *L. plantarum* genes were tested for the presence of NPN-bound LarA*_Lp_* by reacting the samples with LRSA, a reagent that specifically reacts with thiocarboxylic acids (Fig. S2). After resolving the proteins by SDS-PAGE, the protein bands were visualized by Coomassie blue staining (Fig. 3, left), and the LRSA-reactive bands were identified by fluorescence imaging (Fig. 3, right). We compared the intensity of labeling for samples of *E. coli* (pAT035/pAT038) cells that were grown without additive, with 1 mM nicotinic acid, with 1 mM NiCl_2_, and with both 1 mM nicotinic acid and 1 mM NiCl_2_. In addition, we examined a sample of *E. coli* (pAT035) as a negative control and investigated cell-free extracts of *L. lactis* (pGIR112, expressing the four *lar* genes) (2) as a positive control. A band corresponding in size to Strep-tagged LarA*_Lp_* (47.5 kDa) was fluorescently labeled when using samples derived from *E. coli* (pAT035/pAT038) or *L. lactis* (pGIR112) grown in the presence of nickel ions. In the absence of added nickel ions, the LarA band was not labeled, presumably due to a requirement for complete NPN cofactor biosynthesis prior to covalent attachment to LarA in the *E. coli* cells. No band was labeled for *E. coli* (pAT035) that produced LarA and LarB but was incapable of completing NPN cofactor biosynthesis because it lacked LarE and LarC. Analysis of the SDS-PAGE gel by Coomassie staining revealed the presence of the over-expressed LarC*_Lp_* (46.5 kDa), LarE*_Lp_* (30.5 kDa), and LarB*_Lp_* (25.3 kDa) proteins in the appropriate samples. Of additional interest, the relative intensities of several bands were altered in the *L. lactis* lysate, which expresses a different set of genes. Although these results indicate that *E. coli* cells generated NPN-bound LarA*_Lp_*, it does not allow for precise quantification of the labeling.

**Fig 4:**
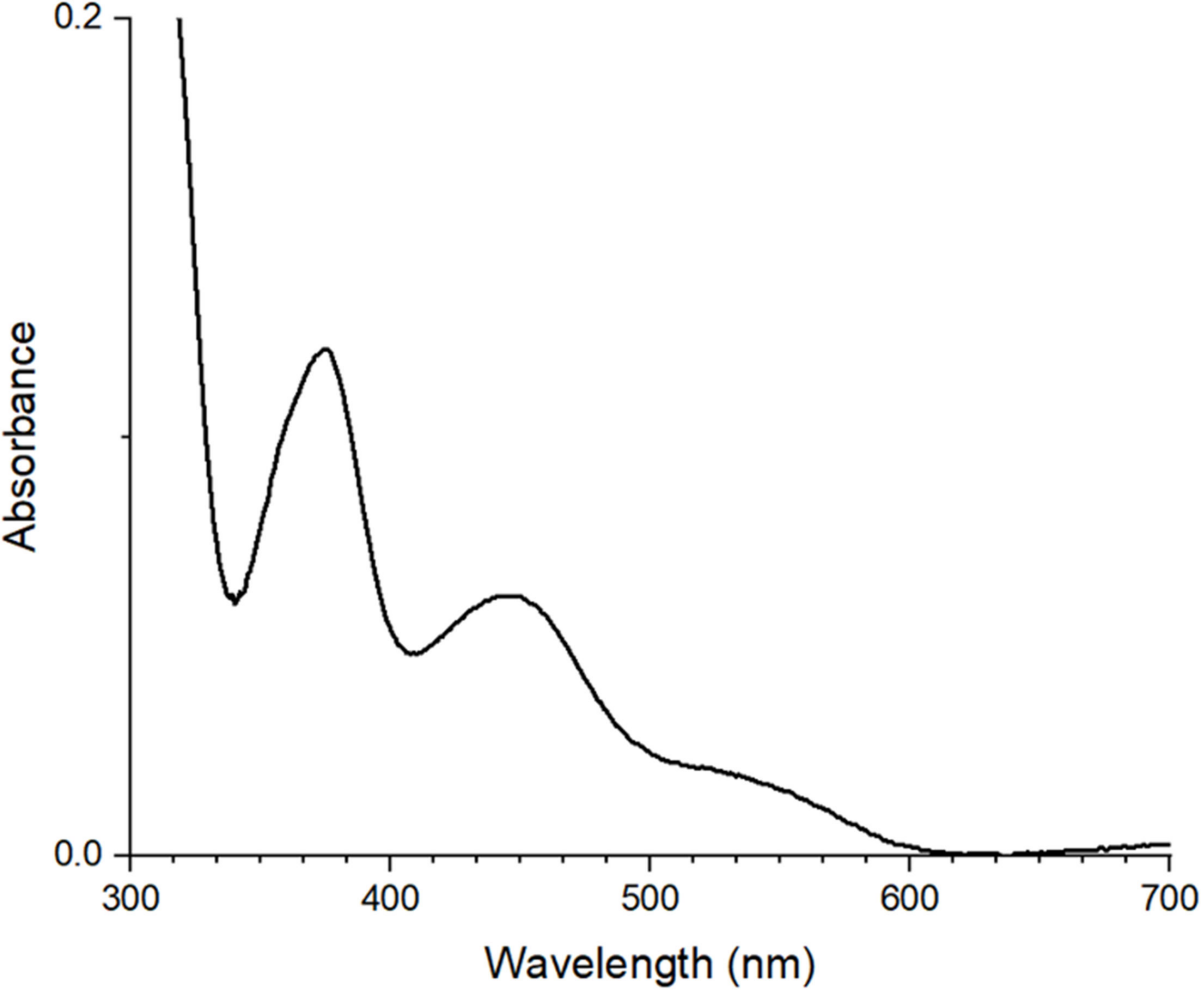
UV-visible difference spectrum obtained by subtracting the spectrum of LarA apoprotein from that of the LarA*_Lp_* holoprotein, derived from the *E. coli* expression system. The absorbance at 280 nm was set to 1.0 for normalization.

As a second method to characterize LarA*_Lp_* that was produced in *E. coli*, we tested for the presence of the NPN cofactor chromophore in the purified sample. The UV-vis spectrum of the enzyme (Fig. 4) revealed electronic transitions at ~380 and 440 nm along with a shoulder at 550 nm; these features are not present in the apoenzyme and are in agreement with prior findings for LarA*_Lp_* purified from *L. lactis* (1). When standardized to the absorption values at 280 nm, the chromophore absorption intensities were approximately half of those reported previously for LarA*_Lp_* (1). This result demonstrates significant, but possibly incomplete, cofactor incorporation and likely also reflects the partial loss of the nickel from the labile chromophore as described earlier (2).

As another method to quantify the NPN cofactor content of the purified protein, we determined its metal content. ICP-OES results obtained within 24 h of purification demonstrated the presence of ~22% ± 2% Ni atoms per LarA*_Lp_* subunit (SD, *n* = 3). The less-than stoichiometric amount of metal associated with the protein is likely due to the instability of nickel within the NPN cofactor, as previously documented for sample that initially exhibited 69% Ni/subunit (2).

**Fig 5:**
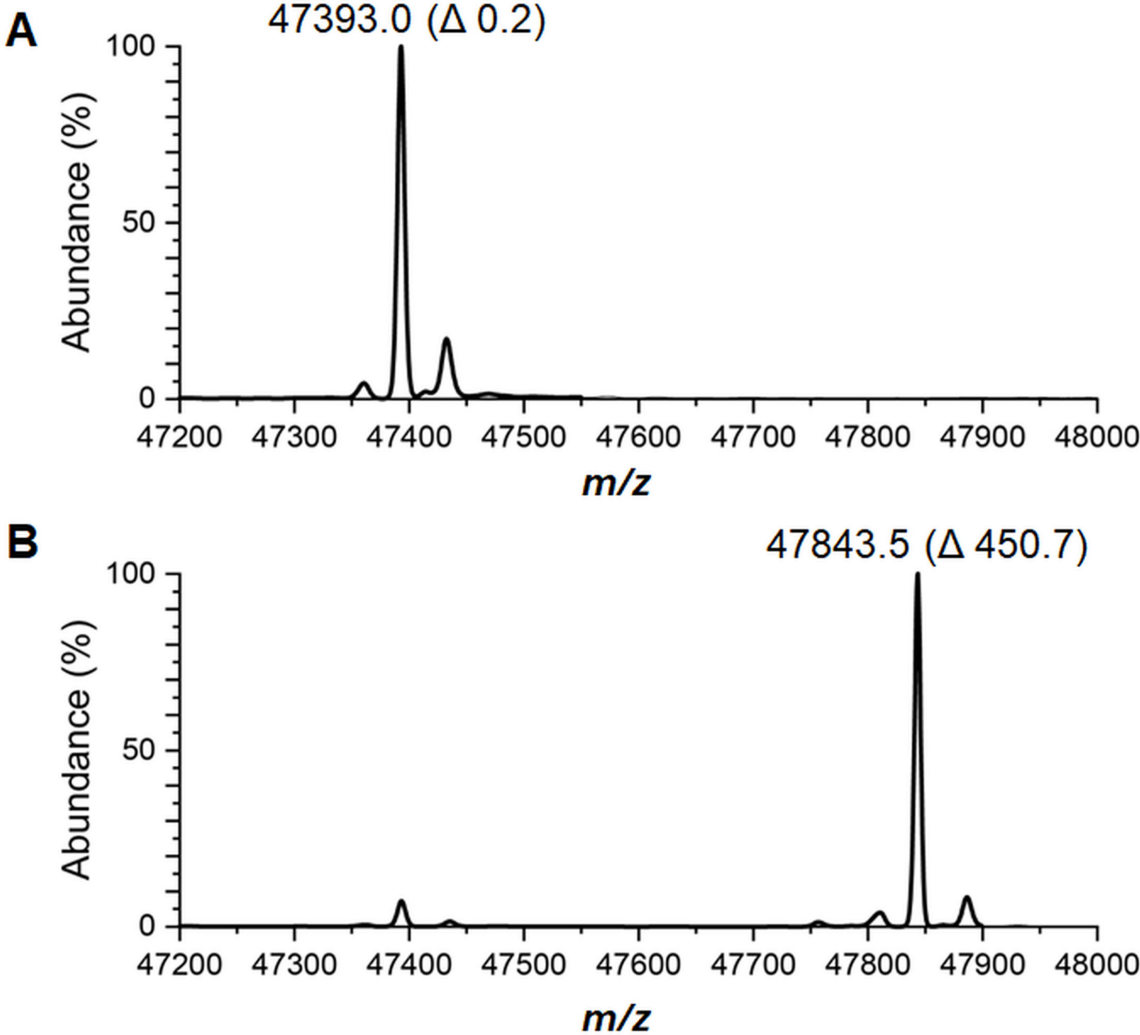
Mass spectra of LarA*_Lp_* purified from the *E. coli* Duet expression system. The mass spectra of (**A**) LarA*_Lp_* apoprotein and (**B**) LarA*_Lp_* holoprotein. The percent abundance is relative to the largest peak. In parenthesis are the mass differences relative to the theoretical mass of the LarA*_Lp_* apoprotein lacking the N-terminal methionine.

A fourth technique to assess the NPN cofactor content of LarA*_Lp_* made use of protein mass spectrometry. Figure 5 shows the results for apoprotein and freshly isolated holoprotein where each sample was shown to be nearly homogeneous and the *m*/*z* values are consistent with the masses expected for the C-terminally Strep-tagged proteins missing their N-terminal methionine residues, with an additional mass difference of 450.7 Da in the holoprotein. This result confirms that a substantial amount of NPN cofactor was covalently bound, presumably to lysine 184, in the *E. coli*-derived *L. plantarum* LarA sample, as previously reported for *L. plantarum* LarA isolated from *L. lactis* (1).

Finally, Lar activity was detected qualitatively in all lysate samples except for the negative control by using the conventional assay method. Furthermore, LarA*_Lp_* that was purified from *E. coli* exhibited a *k*_cat_ of 483 ± 27 s^−1^ and a *K*_*m*_ of 77 ± 12 mM when measured with this assay using L-lactate. Although substantial activity was present, the kinetic parameters reported for the enzyme purified from *L. lactis* were 4,745 ± 544 s^−1^ and 46 ± 20 mM (2), so the heterologous production of this enzyme in *E. coli* resulted in a somewhat compromised activity that may be attributed to decreased cofactor incorporation or rapid inactivation. A newly developed assay for monitoring the activity of *E. coli*-produced LarA*_Lp_* and other racemase samples, described below in a separate section, confirmed the presence of active enzyme. A possible reason to explain the presence of activity in samples that were not labeled by LRSA labeling (from cells provided with only 1 mM nicotinic acid or no additive) (lanes 1 and 6) could be due to the low sensitivity of the LRSA procedure coupled with insufficient levels of covalently bound NPN cofactor on LarA*_Lp_* due to insufficient amounts of bound nickel.

### Use of the Duet system for characterizing NPN-containing LarA homologs

We have successfully substituted genes for several LarA homologs into the pETDuet vector, but here we limit our discussion of this topic to a single illustrative example. *M. elsdenii* is an ecologically crucial rumen bacterium that metabolizes lactate and alleviates rumen acidosis that is generated by a high-grain diet (22). Prior studies had reported the *E. coli* expression of a *larA* homolog from this microorganism followed by purification of the apoprotein (previously named LarAH10, but here termed LarA*_Me_*) (10). Incubation of the apoprotein with biosynthesized NPN provided an enzyme that lacked Lar activity, but it exhibited very low levels of phenyllactate racemase activity (*k*_cat_ of 0.16 ± 0.03 s^−1^ and *K*_*m*_ of 0.4 ± 0.3 mM) as measured by capillary electrophoresis (10). Those results suggested that phenyllactate was not the true substrate of the enzyme, that the protein was only partially loaded with cofactor, or that LarA*_Me_* was rapidly inactivated.

We subcloned the *M. elsdenii larA*-like gene from the previously described pBAD vector (10), substituted it into the pETDuet expression vector, coexpressed it in *E. coli* with genes encoding the NPN-biosynthetic enzymes, and characterized the resulting LarA*_Me_* after isolation using a StrepTactin column. We observed LRSA reactivity of LarA*_Me_* both in cell-free extracts and with the purified protein obtained using the *E. coli* Duet system (Fig. S3). LarA*_Me_* also exhibited the expected weak chromophore spectrum corresponding to the NPN cofactor (Fig. S4). In addition, we showed that ~17% of the purified LarA*_Me_* derived from the *E. coli* system contained nickel, whereas the protein isolated from the *L. lactis* system did not appear to possess this metal ion (10). Of great interest, analysis of the purified LarA*_Me_* by MS revealed several features, some of which are especially instructive for investigators who plan to utilize this method (Fig. S5). A peak of *m*/*z* = 47,936 matches the expected size of the Strep-tagged apoprotein missing its amino terminal methionine residue. A peak corresponding to the intact holoprotein (expected *m*/*z* = 48,387) was not observed, but a peak of *m*/*z* = 48,344 was consistent with loss of nickel (−58.7 Da) and oxidation (+16 Da) of a thiocarboxylate sulfur; such a 408 Da mass difference was reported previously for LarA*_Lp_* (1). Of greatest interest are the two LarA*_Me_* features with *m*/*z* = 48,085 and 48,117. These values are 149 and 181 Da greater than the apoprotein and suggest that dicarboxylated nicotine and dithiocarboxylated nicotine are bound to the protein. Thus, in addition to the potential for losing nickel and undergoing sulfur oxidation, the NPN cofactor in LarA*_Me_* can be unstable due to cleavage of the glycosidic bond that joins the ribose phosphate to the pyridinium ring. Finally, we tested the activity of the *E. coli*-derived LarA*_Me_* using both L-lactate and L-phenyllactate. Whereas earlier studies had failed to detect Lar activity for LarA*_Me_*, we observed an initial rate for conversion of L-lactate to D-lactate of ~2.5 s^−1^, which is three orders of magnitude less than the value of 4,745 s^−1^ reported for LarA*_Lp_* (2). Our analysis of L-phenyllactate racemase activity relied on the ability of D-lactate dehydrogenase to use D-phenyllactate as an alternative (poor) substrate. We measured an initial rate of ~0.25 s^−1^, which is 10% of the value observed for its lactate racemase activity. This result agrees with the previous report of a *k*_cat_ of 0.16 s^−1^ (10). The prior study had quenched the reactions after 5 min, and we found the reactions catalyzed by LarA*_Me_* ceased after about 10 min, consistent with enzyme instability. Overall, our analyses of the *E. coli* purified LarA*_Me_* highlight the lability of the NPN cofactor in this protein and leave open the question of the authentic substrate of this enzyme.

### Investigating the functionality of homologs of potential NPN biosynthetic enzymes

In addition to allowing the generation of active NPN-containing LarA homologs from diverse microorganisms, the Duet plasmid system can be used to test the functionality of genes encoding the homologs of LarB, LarE, and LarC from varied sources. For example, *larC* from *M. thermoacetica* (encoding LarC*_Mt_*), already shown to encode an enzyme with nickel insertase activity (9), was swapped for *larC_Lp_* in pAT038 of the *E. coli* Duet system to create pAT039, and the resulting cells were shown to incorporate the NPN cofactor into LarA*_Lp_* (shown by the Δ450.2 peak) along with a form of the cofactor that was missing the Ni and potentially with a hydroxylated thiocarboxylate (based on the 409.7 Da increase in protein mass) (Fig. S6A). Furthermore, the substitution of LarC*_Mt_* for LarC*_Lp_* in the Duet system allowed the generation of lactate racemase activity. Similarly, swapping the *larC* homolog from *Synechocystis* sp. PCC 6803 (encoding LarC*_Sc_*, UniProt number P72725) for *larC_Lp_* in the pAT038 plasmid yielded cells that derivatized most of its Lar*_Lp_* with bound NPN or a damaged cofactor (increasing the protein mass by 450.2 or 410.7 Da, respectively, Fig. S6B) and exhibited lactate racemase activity, confirming that the cyanobacterial LarC-like protein was capable of inserting nickel into P2TMN and creating a mature NPN cofactor. These findings demonstrate the versatility of the *E. coli* expression system that can be used to test the function of potential biosynthesis pathway enzymes identified only as being sequence homologs.

Showcasing an extreme example of how the Duet system can provide insights into the roles of genes that encode potential NPN cofactor biosynthetic enzymes, we exchanged the *Synechocystis* sp. PCC 6803 homologs of *larB* (encoding LarB*_Sc_*), *larE* (encoding LarE*_Sc_*), and *larC* for the corresponding *L. plantarum* genes in pAT035 and pAT038, while retaining the gene encoding LarA*_Lp_*. The resulting cells were shown qualitatively to possess Lar activity, thus demonstrating that the cyanobacterial genes encode proteins that synthesize the NPN cofactor. Genes encoding LarB, LarE, and LarC are widely distributed in other strains of cyanobacteria, including *Synechocystis* sp. PCC 6803, where they also likely catalyze NPN cofactor biosynthesis. Notably, however, most cyanobacteria lack genes encoding LarA-like proteins. Thus, it is likely that these phototrophs synthesize the NPN cofactor for use in a yet-to-be-identified protein that possesses a sequence and fold distinct from LarA.

### Development of a broadly applicable new assay for NPN-containing LarA homologs

Until recently, assays for NPN-dependent racemases specific to different 2-hydroxyacids required the use of coupling enzymes that reacted with one enantiomer of the distinct substrates (10). A newly developed approach to quantitatively monitor the two enantiomers of a range of 2-hydroxyacids utilizes a capillary electrophoresis approach that included vancomycin in the buffer as a chiral selector (23). Here, we adapted a method using circular dichroism spectroscopy as a broadly applicable technique to monitor 2-hydroxyacid racemases and epimerases. This approach was inspired by similar studies carried out with mandelate racemase that acts on an aromatic 2-hydroxyacid (17).

**Fig 6:**
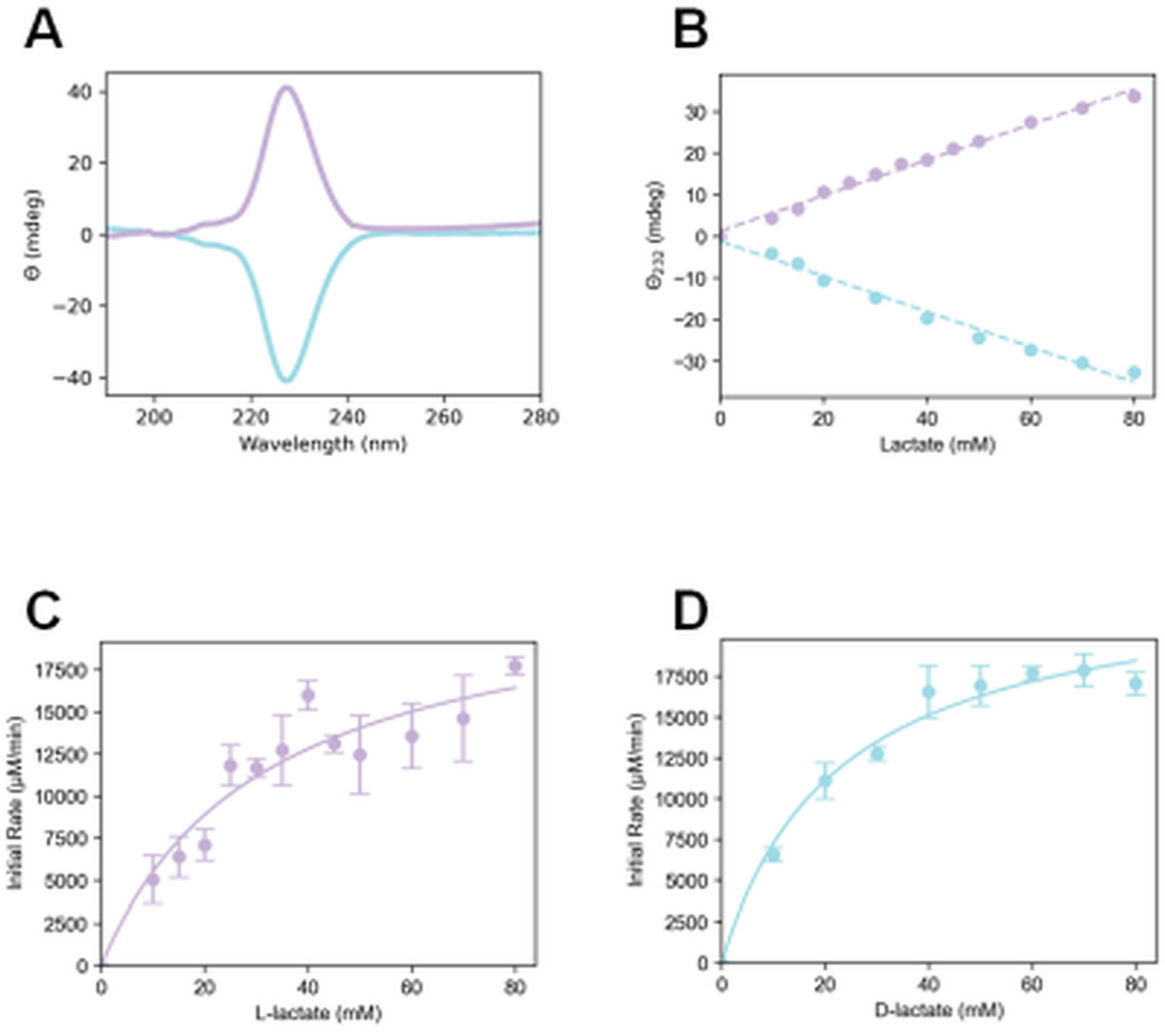
Kinetic analysis of *E. coli*-derived LarA*_Lp_* using a CD assay. (**A**) CD spectra of L- and D-lactate (purple and cyan lines, respectively). (**B**) Standard curves showing the changes in molar ellipticity as functions of L- or D-lactate concentrations. Changes in molar ellipticity versus time provided initial rates at varied concentrations of (**C**) L-lactate and (**D**) D-lactate. The data were obtained at 35°C in phosphate-buffered saline (pH 7.4) using 2.13 µM LarA*_Lp_*.

L- and D-lactate exhibit distinct CD spectra (Fig. 6A), with the maximal difference in ellipticities occurring at a wavelength of 228 nm. To reduce background issues due to absorbance by the substrate, assays were carried out at 232 nm. Standard curves that monitored changes in ellipticity for varying concentrations of lactate yielded straight lines (Fig. 6B). Using the *E. coli*-derived LarA*_Lp_*, the initial rates of racemization were examined as a function of varied concentrations for each lactate enantiomer. Studies with L-lactate provided a *k*_cat_ of 179 ± 25 s^−1^ and a *K*_*m*_ of 32 ± 10 mM, whereas those using D-lactate yielded a *k*_cat_ of 185 ± 12 s^−1^ and a *K*_*m*_ of 23 ± 12 mM (Fig. 6C and D). These rate constants are less than those obtained for LarA*_Lp_* purified from recombinant *L. lactis* cells (*k*_cat_ of 4,745 ± 544 s^−1^ for L-lactate and 1,333 ± 131 s^−1^ for D-lactate) (2), perhaps due to incomplete incorporation of the NPN cofactor or to rapid inactivation of the *E. coli*-derived enzyme. In contrast, the above CD-derived Michaelis constants were comparable to those in the prior report (*K*_*m*_ of 46 ± 20 mM for L-lactate and *K*_*m*_ of 11 ± 4 mM for D-lactate).

**Fig 7:**
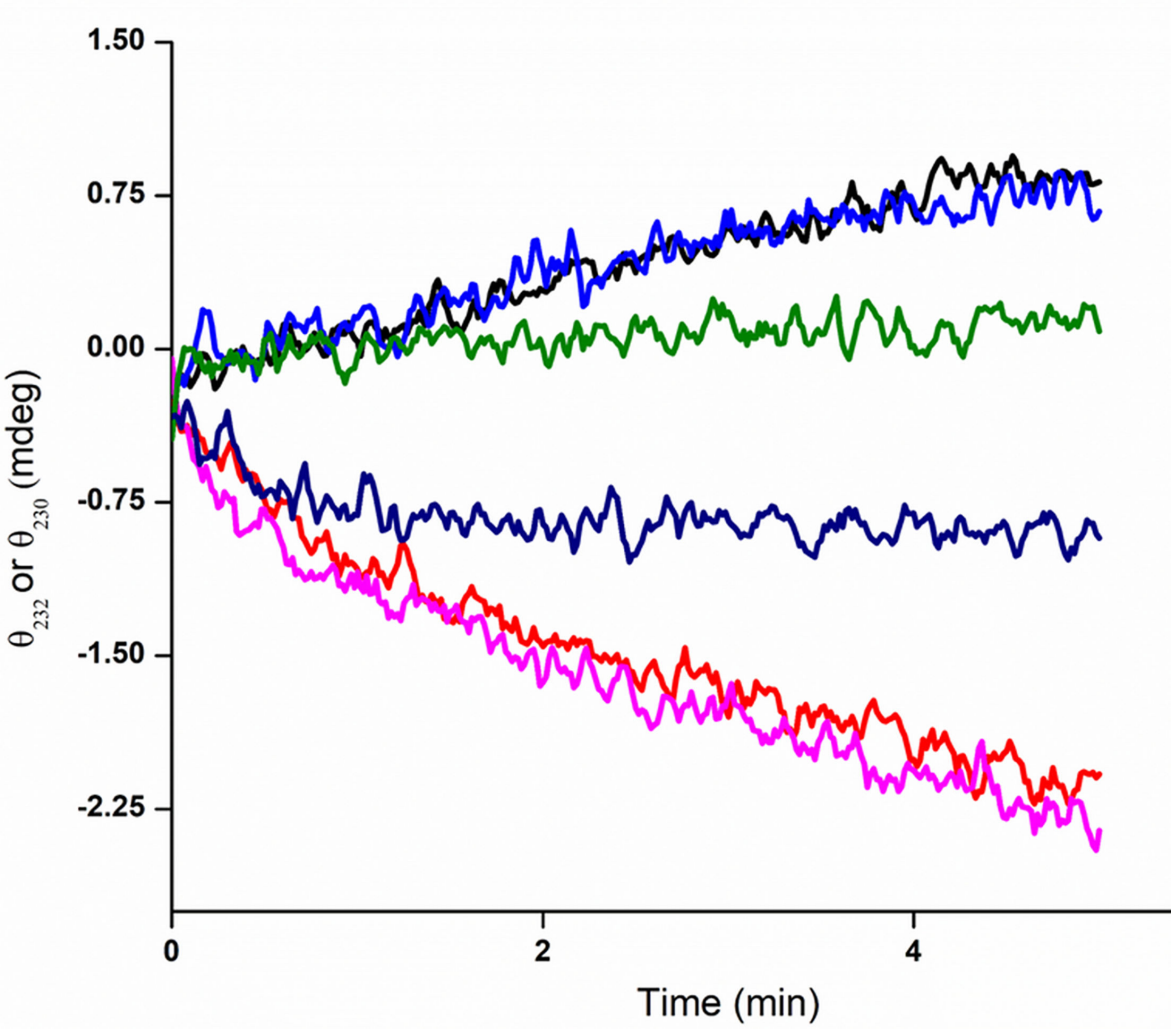
Racemase activity of LarA*_Ip_* using lactate, 2-hydroxybutyrate, and 2-hydroxyisovalerate. Concentrations (25 mM) of D-lactate (black), L-lactate (red), D-2-hydroxybutryate (blue), L-2-hydroxybutyrate (pink), D-2-hydroxyisovalerate (green), and L-2-hydroxyisovalerate (dark blue) in 60 mM phosphate buffer (pH 7.4) were mixed with 100 nM enzyme, and the changes in ellipticities were monitored at 232 nm (lactate and 2-hydroxyisovalerate) or 230 nm (2-hydroxybutyrate) and 55°C.

To demonstrate its versatility and to emphasize its limitations, this assay was applied to LarA*_Ip_* from the thermophile *I. pallida* (10). This enzyme, when purified from *L. lactis*, previously was shown to exhibit lactate racemase activity (*k*_cat_ of 4.7 ± 0.3 s^−1^ and *K*_*m*_ of 0.15 ± 0.04 mM for L-lactate, *k*_cat_ of 8.1 ± 0.5 s^−1^ and *K*_*m*_ of 0.56 ± 0.10 mM for D-lactate). In addition, the earlier study showed this enzyme was inhibited by D-2-hydroxyisovalerate, L-2-hydroxyisovalerate, D/L-2-hydroxyisocaproate, and L-2-hydroxyisocaproate (with *K*_*i*_ values of 0.15 ± 0.02, 1.2 ± 0.2, 2.1 ± 0.3, and 14 ± 4 mM, respectively) (10). Using the CD assay, we confirmed that lactate is a substrate and newly demonstrated that the enzyme also racemizes 2-hydroxybutyrate and, very poorly, 2-hydroxyisovalerate (Fig. 7). Unfortunately, we were not able to define the kinetic parameters for LarA*_Ip_* utilization of these substrates because the assay method lacked sufficient sensitivity; i.e., the changes in ellipticity were too small at low substrate concentrations so that the *K*_*m*_ values were not reliably determined. While performing these studies, we also noted that the signal amplitudes of these substrates were temperature dependent (Fig. S7), with the signal diminishing in intensity at the growth temperature of the microorganism compared to the intensities measured at 25°C. The wavelengths associated with the maximal ellipticities also shift with temperature as most readily seen in the case of 2-hydroxyisovalerate. These points should be considered when designing assay experiments using this approach for other 2-hydroxyacid racemases/epimerases purified from thermophilic microorganisms. The CD spectra of 2-hydroxymandelate (17) and several other 2-hydroxyacids (Fig. S8) demonstrate the potential broad utility of this assay method.

## DISCUSSION

In this work, we have made important progress to overcome two roadblocks that had hindered analysis of NPN cofactor-related proteins from diverse microorganisms. First, the ability to synthesize active NPN-containing LarA homologs encoded by the Duet plasmids in *E. coli*, a genetically tractable microorganism, greatly facilitates functional analysis of these enzymes. In addition, the Duet plasmid system allows for testing the functionality of the non-*L*. *plantarum*-encoded proteins. Second, the circular dichroism assay is broadly applicable to 2-hydroxyacid racemases and avoids the need to develop a distinct coupled enzyme assay for each substrate.

The Duet plasmid system is a significant advance for generating active LarA homologs; however, these cofactor-containing proteins are labile and, even when freshly examined, they may not contain stoichiometric levels of the NPN cofactor. This system potentially could be improved by growing the cells anaerobically, utilizing an *E. coli* strain lacking a nickel exporter (e.g., *rcnA*), or using a strain with altered nickel homeostasis (such as one with a defective NikR) that would lead to continuous importation of the metal ion. The labile nickel pool in *E. coli* was shown to include complexes with four metabolites (oxidized glutathione, histidine, aspartate, and ATP) that function as a reservoir and buffer (24), so perturbations of their levels might allow for enhanced NPN cofactor biosynthesis. Modifications to the growth conditions (e.g., reduction in temperature, provision of NaAD precursors, and supplementation with greater levels of nickel ions) may further improve the extent of activation for the LarA-like proteins. Additional engineering of the strain could boost the production of NaAD in the cell by increasing expression of the genes in the Preiss-Handler pathway (i.e., genes encoding nicotinic acid phosphoribosyl transferase and nicotinate-nucleotide adenylyltransferase) (25). The use of additional compatible plasmids with complementary antibiotic resistance markers from the Duet expression system (14) could allow the introduction of genes encoding other enzymes that could facilitate alternative NPN biosynthesis pathways. For example, one could incorporate *iscS* or other iron-sulfur biosynthetic genes to assist with synthesis of LarE versions containing a [4Fe-4S] cluster (7). Finally, codon optimization of all genes to be expressed in *E. coli* is an important consideration when using the Duet system for production of Lar proteins.

The CD-based assay approach for direct and continuous monitoring of racemase and epimerase activities, described above, is well suited for use with a range of 2-hydroxyacids. This novel approach for monitoring this family of enzymes does, however, have limitations such as when trying to obtain kinetic parameters for enzymes with very low *K*_*m*_ values or when monitoring enzymes obtained from thermophiles. Temperature-dependent CD spectra similar to what we describe have been reported previously for other compounds (26, 27). In selected situations, the continuous monitoring of racemase/epimerase reactions also is possible by use of coupled enzyme spectrophotometric assays, such as by linking lactate racemase with L- or D-lactate dehydrogenase activities (15); however, these coupled assays are typically performed in a discontinuous manner (10). Another discontinuous method to assess these activities uses capillary electrophoresis with the chiral selector vancomycin to separate the enantiomers (23). Together, these assays offer investigators multiple methods to extend our understanding of the activities of NPN-containing enzymes.

## SUPPLEMENTAL MATERIAL

Supplemental Information mbio.03404-24-s0001.pdf Supplemental table and figures.

ASM does not own the copyrights to Supplemental Material that may be linked to, or accessed through, an article. The authors have granted ASM a non-exclusive, world-wide license to publish the Supplemental Material files. Please contact the corresponding author directly for reuse.
