# Supplementary material for: Overcoming barriers for investigating nickel-pincer nucleotide cofactor-related enzymes
Source: mBio. 2024 Dec 16;16(2):e03404-24. doi: 10.1128/mbio.03404-24 (PMC11796402; doi:10.1128/mbio.03404-24)
Supplement: Supplemental Information — Supplemental table and figures. [file mbio.03404-24-s0001.pdf]

Supplemental Information

Overcoming barriers for investigating nickel-pincer nucleotide cofactor-related enzymes

Jorge L. Nevarez,[1] Aiko Turmo,[2] Santhosh Gatreddi,[2] Swati Gupta,[2] Jian Hu,[1,3] and Robert P. Hausinger[2,3]

[1]Department of Chemistry, Michigan State University, East Lansing, Michigan, USA

[2]Department of Microbiology, Genetics, and Immunology, Michigan State University, East Lansing, Michigan, USA

[3]Department of Biochemistry and Molecular Biology, Michigan State University, East Lansing, Michigan, USA

Table S1 List of strains, plasmids, and primers

**Strain, plasmid or primer**	**Characteristic(s) or sequence**		**Source or reference**
**<u>Strains</u>**			
Lc. Lactis			
NZ3900	MG1363 derivative		(1)
E. coli			
DH5α	F– φ80lacZΔ M15 Δ (*lacZYA-argF*) *U169 recA1 endA1 hsdR17* (rK– mK+) *phoA supE44* λ- *thi–1 gyrA96 relA1*		ThermoFisher
BL21 (DE3)	*fhuA2 [lon] ompT gal (λ DE3) [dcm] ΔhsdS* *λ DE3 = λ sBamHIo ΔEcoRI-B int::(lacI::PlacUV5::T7 gene1) i21 Δnin5*		NEB
**<u>Plasmids</u>**			
pETDuet	Amp^r		Novagen
pRSFDuet	Kan^r		Novagen
pAT035	Amp^r	LarA$_{Lp}$ and LarB$_{Lp}$ expression; LarA$_{Lp}$ purification	This study
pAT038	Kan^r	LarE$_{Lp}$ and LarC$_{Lp}$ expression	This study
pAT039	Kan^r	LarE$_{Lp}$ and LarC$_{Mt}$ expression	This study
pAT040	Kan^r	LarE$_{Lp}$ and LarC$_{Sc}$ expression	This study
pETDuet-LarA-Me	Amp^r	LarA$_{Me}$ and LarB$_{Lp}$ expression	This study
pETDuet-LarB-Sc	Amp^r	LarA$_{Lp}$ and LarB$_{Sc}$ expression	This study

pRSFDuet-LarEC-Sc	*Kan*^{r}	$LarE_{Sc}$ and $LarC_{Sc}$ expression	This study
pGIR112		For overexpression of LarA fused with a Strep-tag at the C-terminus from the *larABC1C2DE* operon	(2)
pGIR210-LarAH31	*Chl*^{r}	For overexpression of $LarA_{lp}$ fused with a Strep-tag at the C-terminus from the *larABC1C2DE* operon	This study
<u>**Primers**</u>	The orientation is 5' to 3' in all cases.		
LarALp-Strep_fw	CTTTAAGAAGGAGATATACCATGTCCGTTGCAATTGATTTACCATATGACAA		Subcloning
LarALp-Strep_rv	CCGCAAGCTTGTCGACCTACTTCTCAAATTGTGGATGACTCCAGC		Subcloning
pETDuet_MCS1_fw	CATCCACAATTTGAGAAGTAGCTTAAGTCGAACAGAAAGTAATCGTATTGTAC		Subcloning
pETDuet_MCS1_rv	CATATGGTAAATCAATTGCAACGGACATGGTATATCTCCTTCTTAAAG		Subcloning
LarBLp_fw	GTTAAGTATAAGAAGGAGATATACATATGGCAACCACAGCAGAAATATTACAACAAGTG		Subcloning
LarBLp_rv	CCAGACTCGAGGGTACCTTACATTTGATTGACCATACTAGCTGAGTAGG		Subcloning
<u>pETDuet_MCS2_fw</u>	GTATGGTCAATCAAATGTAAGGTACCCTCGAGTCTGGTAAAG		Subcloning
pETDuet_MCS2_rv	CTGCTGTGGTTGCCATATGTATATCTCCTTCTTATACTTAACTAATATAC		Subcloning
LarELp_fw	CTTTAATAAGGAGATATACCATGGCAACATTAGCAACAAAAAAAGCAACGTTAGTA		Subcloning
LarELp_rv	CTGTTCGACTTAAGCTAGGCGAAAGTGGCCAATTG		Subcloning
pRSFDuet_MCS1_fw	CCACTTTCGCCTAGCTTAAGTCGAACAGAAAGTAATCGTATTGTACA		Subcloning
pRSFDuet_MCS1_rv	GCTAATGTTGCCATGGTATATCTCCTTATTAAAGTTAAACAAAATTATTTC		Subcloning
LarCLp_fw	GTATAAGAAGGAGATATACATATGGGTGCTCAAACACTTTATTTAGACGCTTTTTC		Subcloning
LarCLp_rv	CCAGACTCGAGGGTACCTTACGCCTCCTCATCTAATTGATCTACCG		Subcloning
pRSFDuet_MCS2_fw	GATGAGGAGGCGTAAGGTACCCTCGAGTCTGGTAAAG		Subcloning
pRSFDuet_MCS2_rv	GCGTCTAAATAAAGTGTTTGAGCACCCATATGTATATCTCCTTCTTATACTTAAC		Subcloning
LarCSc_fw	GAAGGAGATATACATATGGGTCTGATCGCC		Subcloning
LarCSc_rv	GACTCGAGGGTACCTTAGCTTTCCGG		Subcloning
pRSFDuet_MCS2_Sc_fw	CTGAGTCCGGAAAGCTAAGGTACCCTCGAG		Subcloning
pRSFDuet_MCS2_Sc_rv	CAAAATAGGCGATCAGACCCATATGTATATCTCC		Subcloning
LarCMt_fw	GAAGGAGATATACATATGAAGATCGCCTATTTTGATTGCTTTAGC		Subcloning
LarCMt_rv	GACTCGAGGGTACCTTAAAATGCTTTCAGTGCACGTGCCGC		Subcloning
pRSFDuet_MCS2_Mt_fw	GCACTGAAAGCATTTTAAGGTACCCTCGAGTCTGG		Subcloning
pRSFDuet_MCS2_Mt_rv	CAAAATAGGCGATCTTCATATGTATATCTCCTTCTTATACTTAAC		Subcloning

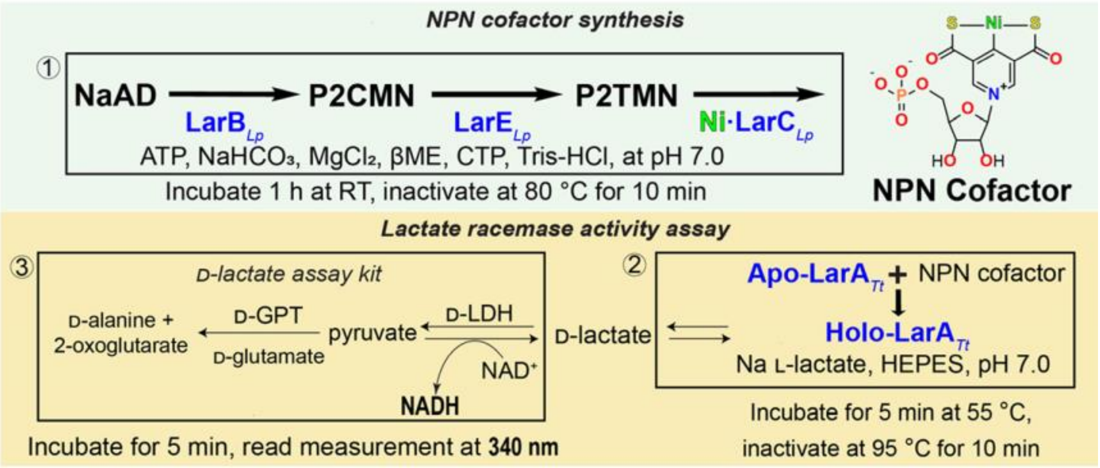

FIG S1 Workflow of the conventional *in vitro* Lar assay. (1) The genes encoding LarB, LarE, and LarC from *L. plantarum* were separately expressed in *E. coli* and the purified proteins were used to convert NaAD via P2CMN and P2TMN intermediates to the NPN cofactor. (2) The cofactor was incorporated into the purified *T. thermosaccharolyticum* LarA apoprotein or into a LarA homolog from another microorganism. (3) The activated LarA was mixed with L-lactate and racemization was assayed spectrophotometrically by quantifying the amount of D-lactate produced based on the reduction of NAD^+ by D-lactate dehydrogenase (D-LDH). D-Glutamate-pyruvate transaminase (D-GPT) and added D-glutamate were used to drive the reaction to completion. βME, β-mercaptoethanol; RT, room temperature.

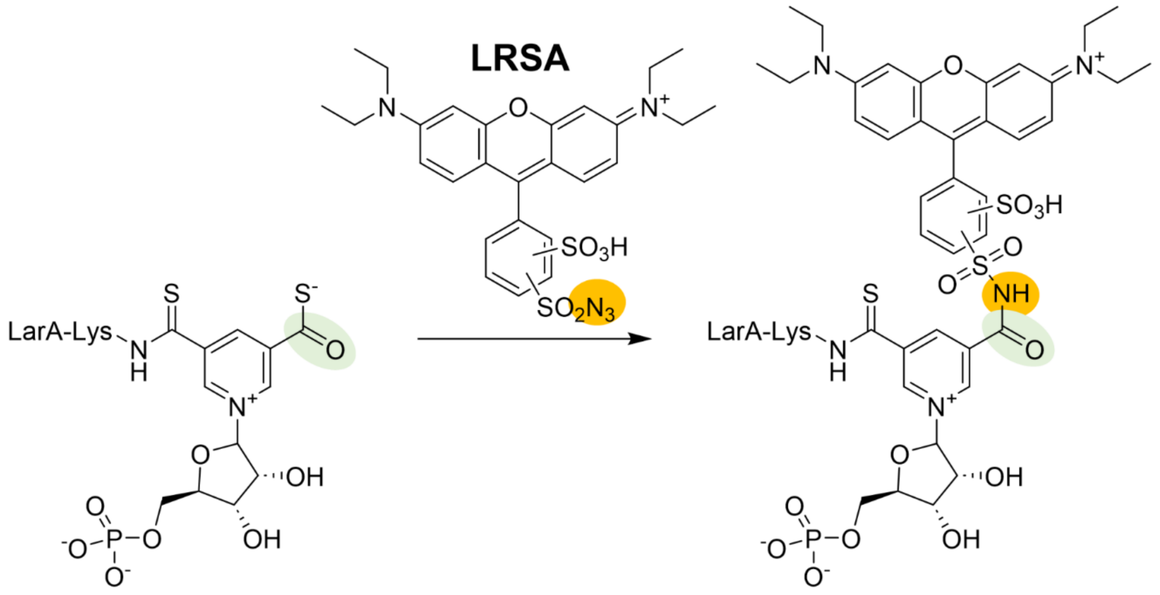

FIG S2 LRSA labeling of the protein bound NPN cofactor. The "click chemistry" reaction between the sulfonyl azide and the thiocarboxylate occurs in the dark at room temperature.

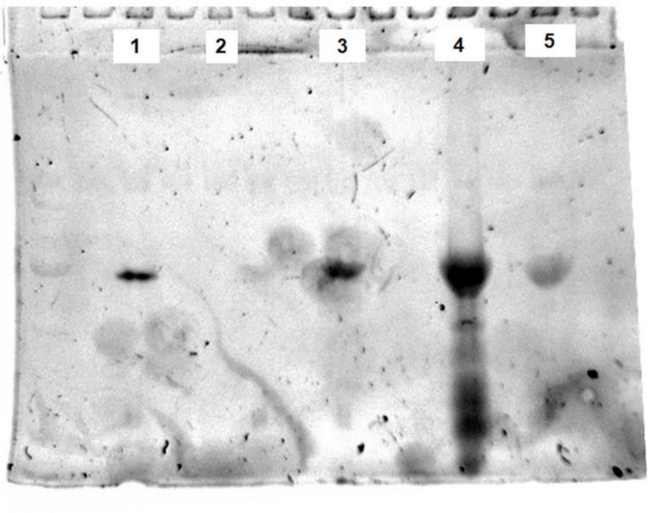

FIG S3 LRSA labeling of LarA$_{Me}$ generated in the *E. coli* Duet system. Samples that were examined for LRSA reactivity include: Lane (1) purified control sample LarA$_{Lp}$ (~130 μg), (2) purified LarA$_{Me}$ apoprotein (~120 μg), (3) lysates containing holoprotein LarA$_{Me}$, (4) isolated holoprotein LarA$_{Me}$ (~180 μg), and (5) the same holoprotein LarA$_{Me}$ (~70 μg).

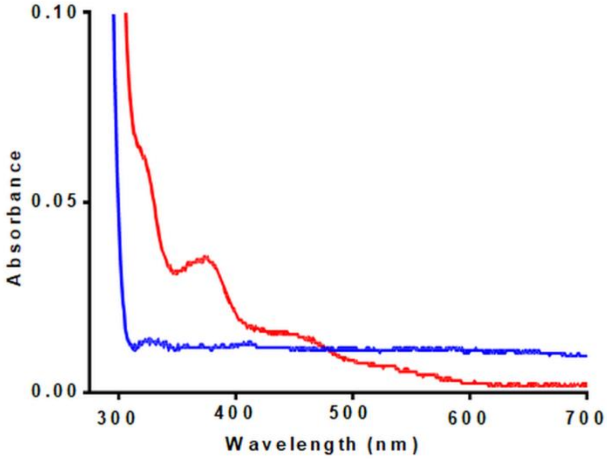

FIG S4 UV-vis spectrum of $LarA_{Me}$ isolated from the *E. coli* Duet system. Spectra are compared for the Lar_{Me} apoprotein (offset blue trace) and holoprotein (red trace) at 12 mg/mL.

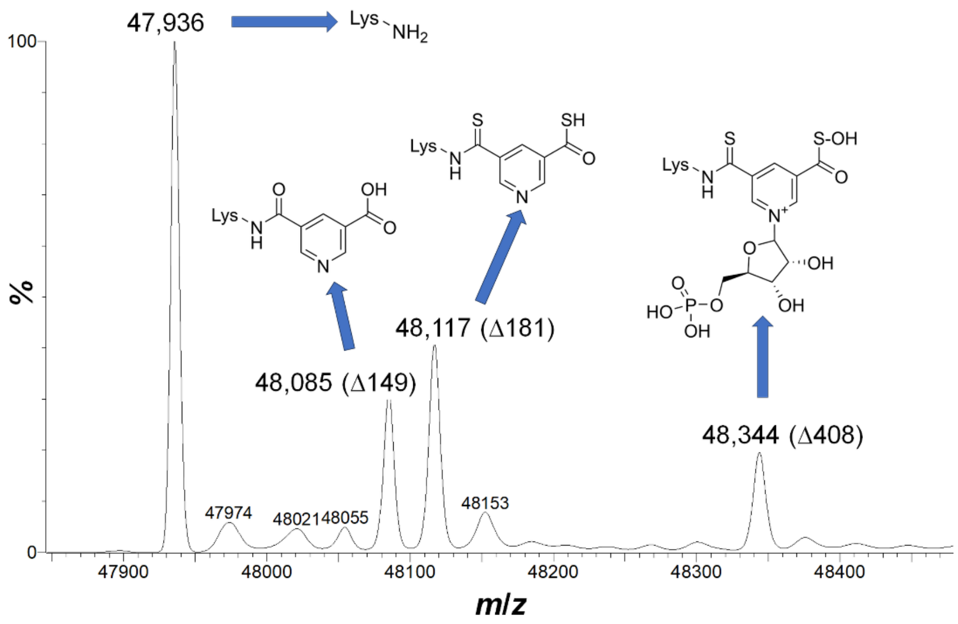

FIG S5 Mass spectrum of $LarA_{Me}$ purified from the *E. coli* Duet expression system. Potential structures matching each feature are illustrated, where Lys is a side chain of the protein.

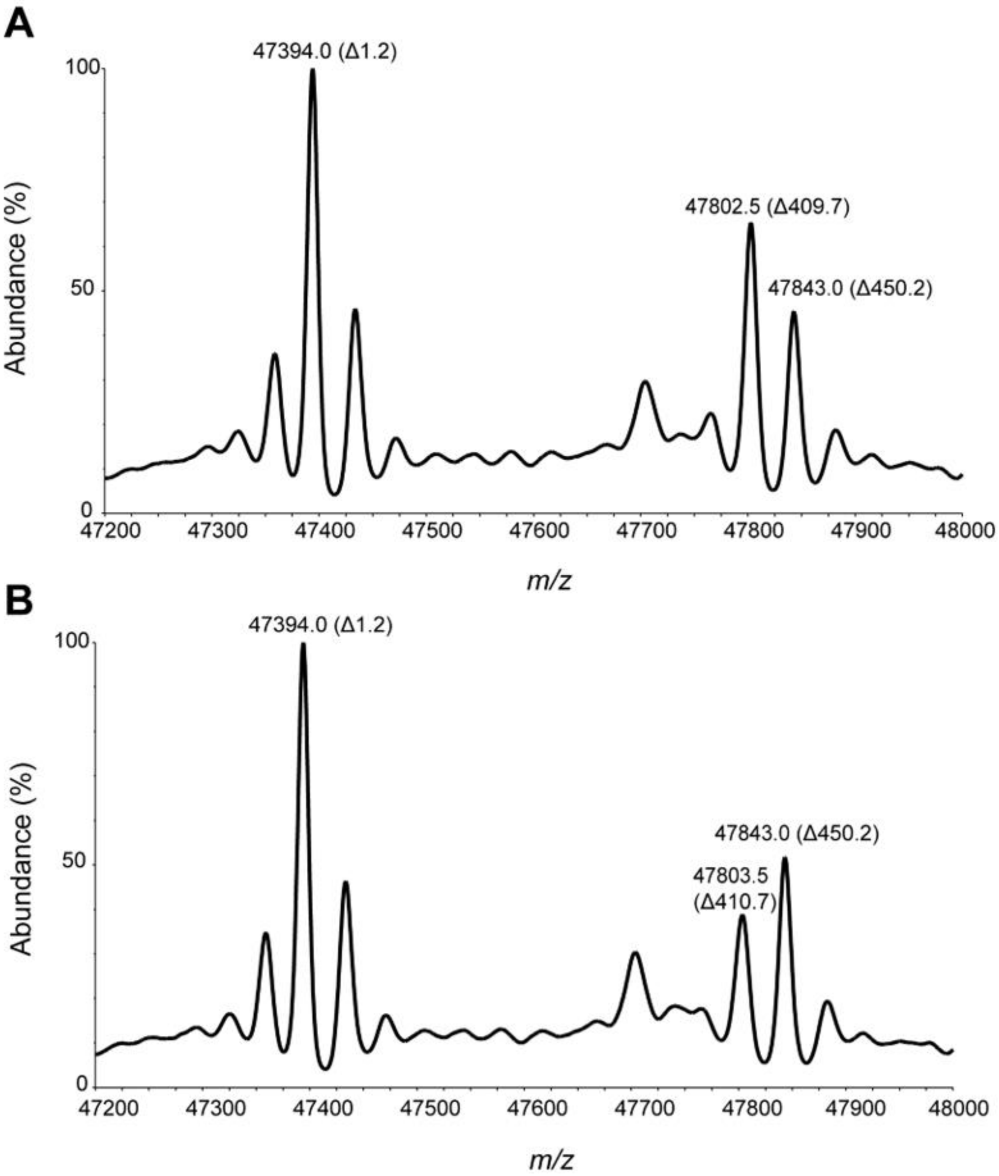

FIG S6 Mass spectrum of $LarA_{Lp}$ purified from the *E. coli* expression system with the gene encoding $LarC_{Lp}$ replaced by genes encoding (A) $LarC_{Mt}$ and (B) $LarC_{Sc}$. The percent abundance is relative to the largest peak and values in parentheses are the mass differences to the theoretical mass of $LarA_{Lp}$ apoprotein lacking the N-terminal methionine.

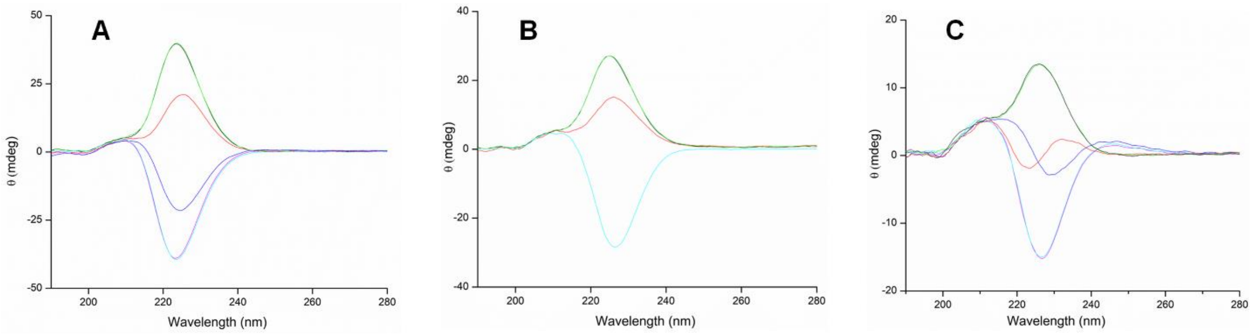

FIG S7 Temperature dependence of the ellipticity for lactate, 2-hydroxybutyrate, and 2-hydroxyisovalerate. (A) Ellipticity of 25 mM L-lactate at 25 °C (black), 55 °C (red), and returned to 25 °C (green) and of 25 mM D-lactate treated similarly (cyan, blue, and magenta). (B) Ellipticity of 25 mM L-2-hydroxybutyrate at 25 °C (black), 55 °C (red), and returned to 25 °C (green) and of 25 mM D-2-hydroxybutyrate at 25 °C (cyan). The signal from D-2-hydroxybutyrate also decreases in intensity at higher temperature (not shown). (C) Ellipticity of 25 mM L-2-hydroxyisovalerate at 25 °C (black), 55 °C (red), and returned to 25 °C (green) and of 25 mM D-2-hydroxyisovalerate treated similarly (cyan, blue, and magenta). All spectra were collected using 60 mM phosphate buffer at pH 7.4.

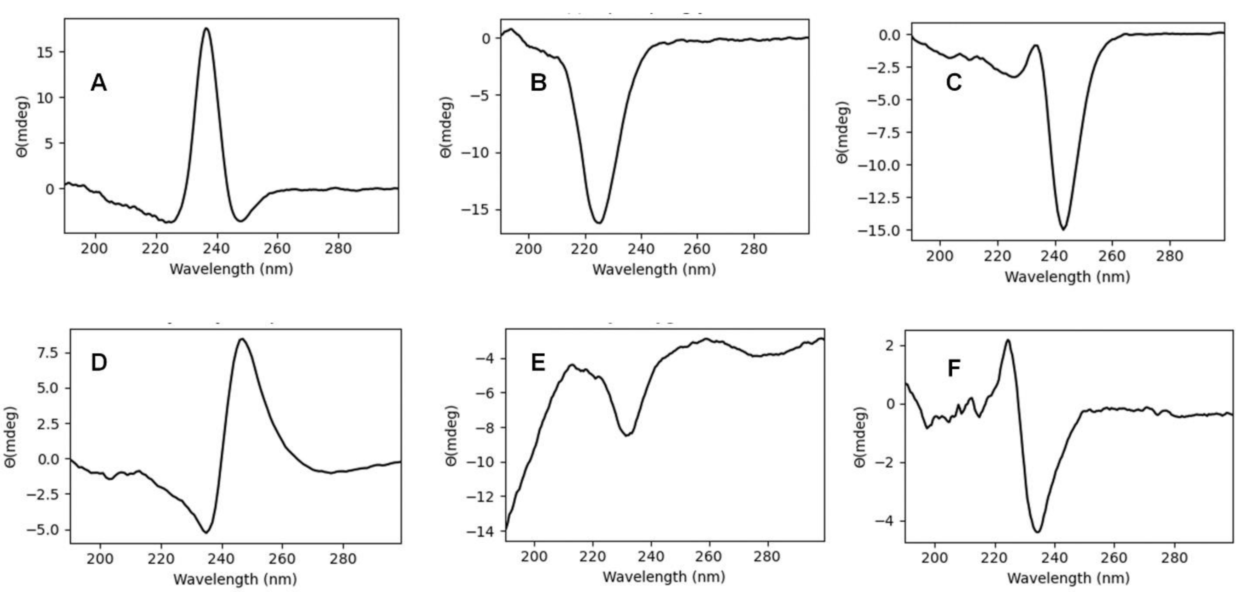

FIG S8 CD spectra of selected other 2-hydroxyacids. (A) L-Malate. (B) D-Phosphoglycerate. (C) L-2-Hydroxy-3-methylbutyric acid. (D) L-2-Hydroxyisocaproate. (E) D-2-Hydroxyglutarate. (F) D-Gluconate. The samples contained 10 mg of hydroxyacid in water and were examined at 35 °C.

REFERENCES

1. de Ruyter PG, Kuipers OP, de Vos WM. 1996. Controlled gene expression systems for *Lactococcus lactis* with the food-grade inducer nisin. Appl Environ Microbiol 62:3662-3667.
2. Desguin B, Goffin P, Viaene E, Kleerebezem M, Martin-Diaconescu V, Maroney MJ, Declercq J-P, Soumillion P, Hols P. 2014. Lactate racemase is a nickel-dependent enzyme activated by a widespread maturation system. Nat Commun 5:3615.
